# Long-Lasting Neural Circuit Dysfunction Following Developmental Ethanol Exposure

**DOI:** 10.3390/brainsci3020704

**Published:** 2013-04-29

**Authors:** Benjamin Sadrian, Donald A. Wilson, Mariko Saito

**Affiliations:** 1 Department of Child and Adolescent Psychiatry, New York University Langone School of Medicine, One Park Avenue, Eighth Floor, New York, NY 10128, USA; E-Mail: dwilson@nki.rfmh.org; 2 Nathan Kline Institute, 140 Old Orangeburg Road, Orangeburg, NY 10962, USA; E-Mail: marsaito@nki.rfmh.org; 3 Department of Psychiatry, New York University Langone School of Medicine, One Park Avenue, Eighth Floor, New York, NY 10128, USA

**Keywords:** alcohol, FASD, neural circuit, brain development, excitation/inhibition balance, neurodegeneration

## Abstract

Fetal Alcohol Spectrum Disorder (FASD) is a general diagnosis for those exhibiting long-lasting neurobehavioral and cognitive deficiencies as a result of fetal alcohol exposure. It is among the most common causes of mental deficits today. Those impacted are left to rely on advances in our understanding of the nature of early alcohol-induced disorders toward human therapies. Research findings over the last decade have developed a model where ethanol-induced neurodegeneration impacts early neural circuit development, thereby perpetuating subsequent integration and plasticity in vulnerable brain regions. Here we review our current knowledge of FASD neuropathology based on discoveries of long-lasting neurophysiological effects of acute developmental ethanol exposure in animal models. We discuss the important balance between synaptic excitation and inhibition in normal neural network function, and relate the significance of that balance to human FASD as well as related disease states. Finally, we postulate that excitation/inhibition imbalance caused by early ethanol-induced neurodegeneration results in perturbed local and regional network signaling and therefore neurobehavioral pathology.

## 1. Overview of FASD and Its Impact on Society

Early ethanol exposure in humans can result in a range of measurable neurobehavioral deficits, clinically summarized as fetal alcohol spectrum disorder (FASD). FASD can be detected in afflicted offspring from early childhood with symptoms that often persist throughout adulthood. Reported characteristics of FASD include impaired cognitive performance, adaptive behavior disorders, deficits in sensory processing as well as executive functions of assessment and decision making, as well as a higher risk of seizures [1,2,3,4,5]. Fetal alcohol syndrome (FAS) remains a designation of the most severe condition caused by early alcohol exposure that includes facial abnormalities and severe cognitive and/or behavioral deficits. Up to 1 in 100 children born in the U.S. and Canada are diagnosed with FASD [6,7], making it one of the leading causes of mental deficiency in western nations [8]. The first scientific record connecting disorders in offspring with maternal alcohol abuse was made over a century ago [9]. Although FASD is completely preventable, and the teratogenic effects of fetal alcohol exposure have gained public consciousness, the rate of FASD diagnoses have not decreased since the term fetal alcohol syndrome (FAS) was coined forty years ago [10]. This is largely because those at greatest risk are offspring to women who abuse alcohol compulsively, in a behavioral pattern that is less amenable to centralized education programs and warning labels [11]. Still, studies on preventative education and intervention have shown promise for improved awareness and decreased risk-taking across a more general population [12,13,14,15]. Promoting educational awareness and intervention as well as translational research programs is not only urgent from a medical and ethical standpoint, but is economically advantageous as well. Annual costs of FASD to society in the United States are currently estimated at approximately $5 billion per year [16,17]. Interestingly, a previously examined connection of paternal alcoholism to fetal development disorders [7,18,19] has been revitalized by recent studies providing evidence for epigenetic alterations [20], that may be carried through the male germline for generations [21]. This early evidence suggests that alcohol effects on fetal development may not only be caused by maternal alcohol consumption, but may be influenced by the father’s exposure as well. Thus, an understanding of, and treatments for alcohol addiction in both men and women may be the most efficient preventative treatment for FASD.

Neuroprotective agents hold a prophylactic potential for pregnant women diagnosed with alcoholism to significantly reduce the rate of children born with diagnosable FASD. However, potential drugs thus far found effective in limiting ethanol-induced neurodegeneration, such as lithium [22,23,24,25] have reported detrimental effects of their own on the developing fetus [26]. A lower risk approach of supplementing diets with antioxidants [27,28,29], taurine [30], niacin [31], GM1 gangliosides [32] or resveratrol [33], all of which have been shown to limit ethanol-induced neurodegeneration, would still require compliance from expecting mothers that abuse alcohol compulsively. Another problematic group are women who binge-drink without yet knowing they are pregnant, and are therefore not aware of a risk in the first place. Therefore, the inevitable stream of newly affected offspring substantiates a need for a greater understanding of the mechanisms of alcohol-induced neuropathologies in order to generate therapeutic strategies of damage control and/or reversal. Here, we present a hypothesis that many neurologic symptoms of FASD may be explained as manifestations of imbalance between excitatory and inhibitory neuronal activity in local and regional networks, which would normally support the affected functionality. We first provide a primer on the background information and methodologies typically utilized to examine neuronal circuit function, followed by evidence from recent studies in models of FASD that support our hypothesis.

## 2. Assays of Basic Neural Circuit Function

There is a diverse array of assays for neural circuit function and dysfunction, with a wide range of spatial and temporal sensitivity. For example, single-unit electrophysiology allows examination on a millisecond time scale of the firing pattern of one or a few individual neurons within a selected brain region. In some cases, by analyzing firing patterns or by using juxtacellular labeling, the recorded cell can also be identified as a member of a specific cell class, such as a pyramidal cell or interneuron. Thus, a great deal of information regarding how an individual cell or cell class functions can be extracted with high temporal precision using single-unit techniques. However, it is increasingly apparent that neurons function in ensembles and networks, thus sampling an individual of a population may provide limited information regarding how a brain region functions as a whole. In contrast, techniques such as immunohistochemistry for immediate early gene products, such as c-fos, c-jun, arc, or zif allow assaying the activity of essentially all neurons within a brain region (or multiple regions), with resolution at the cellular level. These immediate early genes are “switched-on” when a neuron is activated, for example by sensory input, and the gene protein product can be detected by immunohistochemistry. When combined with double-labeling techniques, the identity of the activated cells can also be determined, for example as a GABAergic inhibitory interneuron or an excitatory pyramidal neuron expressing calcium-calmodulin kinase II. However, the tremendous spatial and cellular resolution comes at a cost. Immunohistochemical techniques provide individual snapshots of activity, and generally represent activity that is summed over many minutes. Thus, any temporal structure in the signal is lost.

This trade-off between temporal and spatial resolution is apparent in essentially all functional neural assays. Electroencephalograms (EEG; recorded on the scalp) and local field potential (LFP; recorded with depth electrodes) recordings provide good temporal resolution but limited spatial resolution. Both of these techniques measure the activity of many thousands or millions of neurons relatively near the electrode. Given a similar number of active neurons, the greater the synchrony in activity of these neurons (generally synaptic potentials) the larger the recorded voltage fluctuations. Neural activity is metabolically expensive which provides another way to monitor brain physiology. Assays that measure metabolic activity or changes in oxygenated blood flow (e.g., ^14^C-2-deoxygucose uptake, functional magnetic resonance imaging (fMRI), or optical imaging of intrinsic signals) as indirect metrics of neural activity, share similar spatial/temporal trade-offs though generally with much reduced temporal precision than EEG or LFP recording. 

Finally, neural activity is ultimately dependent on synaptic transmission, and a variety of methods are available for direct assessment of relative synaptic strength. This can include either extracellular evoked potential recordings in simple monosynaptic circuits that follows direct electrical stimulation of afferent fibers, or intracellular recordings of spontaneous and evoked synaptic potentials/currents in individual neurons. These techniques can be performed either *in vivo* or *in vitro*. Assessing synaptic strength allows assessment of the state of synaptic function within an identified circuit, and also allows assessment of how plastic those synapses are; for example, by using long-term potentiation (LTP) and long-term depression (LTD) stimulation protocols.

Any of the above techniques can be used to assay neural circuit function and detect changes from that function. All of these have been used to examine the long-term consequences of developmental ethanol exposure in humans and/or non-human animals including singe-units [34], local field potentials [23,35], EEG and evoked potentials [36,37], intrinsic optical imaging [34,38], fMRI [39,40], and synaptic physiology [23].

### 2.1. Background of Neural Circuit Function

The analytical techniques described above can demonstrate disruption of normal neural circuit function following early ethanol exposure. In this section we briefly review what is known about how normal neural circuits work to encode information, and describe the roles of circuit components in said functions. This will provide a background for interpreting early ethanol induced changes in function and help identify critical circuit components impacted by early ethanol-induced dysfunction. In many regions of the brain, information is contained in both the spatial location of the active neurons (e.g., topographical organization) and the temporal structure of their spike trains. 

#### 2.1.1. Spatial Information Coding

The brain processes and stores information in order to guide behavior. Nearly all sensory systems have some form of spatial organization related to the stimulus energies being processed, including retinotopic, somatotopic, tonotopic and odotopic maps in the visual, somatosensory, auditory and olfactory systems respectively. There may also be spatial organization of activity in the gustatory cortex based on the quality of the tastants producing a gustotopic map [41]. Spatial organization of information allows for interactions between neurons conveying highly similar information. For example, lateral inhibition between neighboring neurons responding to similar inputs (e.g., similar sensory inputs, similar memories, similar actions) can enhance the contrast between those inputs by shifting the activity to one or the other and enhancing acuity/precision of the sensation/memory/action. Lateral inhibition of this sort is much more effective if there is spatial organization in the neurons collectively encoding the information as a composite of parts, because inhibition would be most robust where it is needed most - between those neurons with very similar response properties. 

The ontogeny of spatial neural organization and spatial maps are heavily dependent on early experience. The formation and maintenance of neural circuits is activity-dependent, where active circuits are generally fortified and less active or less effective circuits are pruned. Furthermore, early experience can affect the structure and survival of both principal neurons and interneurons, thus even if neural circuit architecture was unaffected by early experiences, loss or impairment of interneuron function disrupting lateral inhibition could impair normal spatial functioning and acuity of the circuit.

#### 2.1.2. Temporal Information Coding

In addition to the identity or location of active neurons, information encoding is heavily dependent on the temporal structure of their activity. One form of temporal structure is an increase or decrease in activity, but beyond simple increases and decreases in firing rate of individual neurons, the patterning of neural activity between neurons is also important. This can easily be imagined in systems like motor control regions where specific muscle groups, controlled by specific motor neurons, must be activated in specific temporal sequences in order for the correct movement to occur. The same dependence on timing is true in all neural circuits. 

In addition to activity sequence, the synchrony of activity in disparate neurons is also critical to information processing. Temporal summation properties of neural dendrites means that the more synchronous pre-synaptic inputs are, the more effective they will collectively be in driving the post-synaptic neuron. An indication of neural synchrony can be gained from examining EEG and LFP recordings. The more synchronous neurons are within a region, the greater the sum of extracellular currents and resulting local potential. This activity often emerges as oscillations in the EEG or LFP. The strength of the oscillatory waves is influenced by the number of cells in the involved ensemble, the extent of synchrony in their activity, the number and strength of excitatory synapses connecting them, and the strength of synaptic inhibition. Oscillations can also be influenced by the biophysics of neural membranes, with some cells more tuned to specific frequencies than others due to the kinetics of the ion channels they express. Oscillations of this type have been described throughout the mammalian brain and often fall into a few bands of frequencies. Some of the more commonly studied oscillation frequency bands are delta (typically 0.3–4 Hz), theta (5–15 Hz), beta (15–35 Hz) and gamma (35–90 Hz), though the specific frequency range labels vary between labs. 

Oscillatory activity has several consequences [42]. First, since it derives in part from neural synchrony, information transfer between regions can be enhanced if their oscillations are coherent. The temporal correlation in activity between two brain regions measured by LFP and fMRI is often used as a metric of functional connectivity. This coherence can also help unite information (e.g., stimulus features) contained in scattered neurons or even scattered brain regions to each other (e.g., creating an object). Finally, a patterned synchronous input to target neurons is the most effective way of inducing synaptic plasticity such as LTP. Neurons that fire together, wire together [43]. Oscillations can assist and promote the “firing together” component of that equation.

The temporal structure of neural activity is an important factor in information encoding, information transfer, neural plasticity and the binding of disparate neural activity. Importantly, the balance of synaptic excitation and inhibition in a circuit plays a crucial role in shaping spike train temporal structure and network oscillations. For example, experimental manipulations of synaptic inhibition influence both the magnitude and the frequency of oscillatory activity. In the extreme, reductions in synaptic inhibition can result in epileptiform activity—a pathological oscillation.

### 2.2. Early Circuit Function and Early Ethanol Exposure: A Hypothesis

As described below, work from our lab and others have demonstrated that developmental ethanol exposure results in a long-lasting disruption of normal neural circuit function, assessed with many different techniques. Neural circuit dysfunction leads to impaired cognition and behavior. In this review we hypothesize that the long-term effects of developmental ethanol exposure on neural circuit function are due in part to a deficit in inhibitory interneuron development, and thus a disrupted excitatory/inhibitory balance. Disruptions in excitatory/inhibitory balance have also been recently hypothesized as major contributors to cognitive decline in Alzheimer’s disease [44], autism spectrum disorder [45,46], schizophrenia [47,48] and attention-deficit hyperactivity disorder [49]. The remainder of this review will outline the evidence in support of this hypothesis.

## 3. Animal Models of Early Alcohol Exposure

To examine early ethanol-induced effects on neuronal circuit function, rodent models have proven indispensible. Animal paradigms of ethanol delivery have developed over the past four decades toward dissecting the etiology of neurobehavioral deficits analogous to those observed in human FASD [50]. Both chronic and acute alcohol exposure models have been utilized extensively, and each provides particular neuroanatomical and neurobehavioral effects for the study of various pathologies [51,52,53]. Each model has developed strengths of specialization in the pathogenicity they generate, since the developmental stage of alcohol insult is a key determinant of the experimental outcome [50]. No single animal model has been found to reproduce all known deficits observed in human FASD [54]; therefore, the appropriate model to use depends on the specific topic of investigation. Our summary here largely draws from studies utilizing acute developmental ethanol exposure. By comparison, chronic exposure models are indeed more commonly utilized, and are more reality-based in that they more accurately simulate habitual drinking patterns known to occur in affected human populations [55,56]. We however, highlight specific purposes for which acute exposure possesses key advantages, particularly for examining long-lasting effects of exposure on neural circuit function from specific developmental time points of insult.

Acute ethanol effectively narrows the window of toxin exposure to a specific developmental period. When studying the direct effects of early ethanol exposure on brain development and long-term neuronal function, acute exposure minimizes many confounds inherent to a chronic exposure model. Specifically, protracted ethanol toxicity during chronic exposure cumulatively impacts other body systems that support brain development. For example, chronic alcohol exposure during gestation and lactation can negatively impact kidney function [57] and decrease fetal organ mass ratios [58]. For purposes of neural circuit examination these circumstances perturb the interpretation of causality, because the source of neurotoxicity can either be attributed to the direct interaction of alcohol, or alternatively as a byproduct of metabolic and/or systemic attrition from prolonged alcohol exposure (most likely a combination of these stressors). 

Acute ethanol exposure on the contrary provides a more discrete temporal window. A major drawback of acute ethanol exposure is its translation to a real-world model of FASD. Most acute “binge” exposure models generate an excessively high transient peak blood alcohol content often well above 200 mg/dL, which is already the equivalent of a medium build woman having over ten drinks in a five hour period [59]. Additionally, when early postnatal pups are used in human third trimester equivalent acute studies, the direct subcutaneous injection or feeding of ethanol into neonatal pups does not account for buffering effects of the mother’s placenta nor for maternal alcohol metabolism of the administered dose, which is reported to occur in pregnant rats at an hourly rate of approximately 175 mg/kg [60]. Despite these nuances, the acute exposure model is a valuable tool specifically for tweezing out the immediate molecular mechanisms and cellular consequences of ethanol-induced neurotoxicity in the developing brain, as well as for physiological analyses of neuronal circuit function that includes long-term functional outcomes. This is because the timing of alcohol insult is a major influence on the deficits produced [61]; therefore, temporal precision and a reliable window of toxin exposure allows us to draw developmental correlates between neurobehavioral consequences of alcohol and the specified stage of exposure.

The timing of each established FASD animal model has a neurodevelopmental equivalent to human gestation, which are not necessarily aligned by embryogenesic stage [54]. The most common acute model system used is early postnatal ethanol exposure (within the first two weeks after birth), because it correlates with the vital third trimester of human embryonic brain development [62]. This period witnesses the so-called “brain growth-spurt”, in which synaptogenesis and synaptic refinement flourishes, and therefore yields neurobehavioral consequences of ethanol exposure functionally linked to plasticity-related mechanisms. For the study of acute ethanol-induced effects on circuit function this period also has an advantage of arriving later in the sequence of brain development. It therefore has a lower potential for confounded outcomes that might otherwise occur through stochastically altered progressions of progenitor cell populations. 

## 4. Findings of Neuropathology in Acute Ethanol Exposure Animal Models of FASD

Effects of acute alcohol exposure can be examined during the earliest developmental stages by administering ethanol to the mother during gestation (human first and second trimester equivalents), or at a later developmental stage by direct administration to neonatal pups (human third trimester equivalent). Methods of ethanol delivery include water supplementation, ethanol vaporization, intubation/gavage delivery, or direct subcutaneous injection. In each case a peak blood alcohol concentration over 200 mg/dL is typically achieved, while 50 mg/dL is sufficient to elicit immediate activation of neurodegenerative cascades [63] with significant reductions in brain mass reported above 150 mg/dL [64]. Acute exposure schedules vary between studies, but are classically defined as a single day of exposure. Models with extended exposures ranging up to six successive days have also been utilized with the consideration it targets the same specific stage equivalent of neurodevelpoment. Administered ethanol metabolism varies between species and by mode of exposure. However, as an example of metabolic clearance rate, blood alcohol levels of subcutaneously injected ethanol in early postnatal mice drop within eight hours after administration below levels reported to cause measurable neurodegeneration [60,63]. Therefore, acute exposure limits ethanol-induced toxicity within a temporal confine. The functional impact of ethanol toxicity however, is not necessarily restricted to this same short period. Many studies have demonstrated how acute ethanol exposure can produce long-lasting neuropathologies into early adulthood, including alterations in: neurogenesis [65], neuroanatomy [66,67], neurobehavior [68,69], synaptic physiology and LTP [23,70,71,72], hippocampal spine density [73], and sensory-evoked physiology [35]. Many of these reported defects occur even when exposure lasts only a single day. The concept of a perpetual neuropathology initiated by limited events early in development is one of the most profound contributions from acute ethanol studies, because they illustrate how fatalistically delicate particular stages of neural development truly are in specific brain regions, and how plastically resilient other regions have proven to be.

### 4.1. Neuroanatomical Effects of Acute Ethanol Exposure

One of the most notable effects of early ethanol insult is a devastating wave of neurodegeneration in the developing brain immediately following exposure. Multiple studies have reported a decrease in mass and volume of specific brain regions [74] and/or a decrease in specific cell population numbers [75] as a result of early ethanol exposure. Interestingly, there are suggestions of sexually dimorphic impacts of early ethanol on neurodegeneration, neuronal morphogenesis and neurogenesis [66,76,77]. A single day of acute ethanol exposure in early postnatal mice is sufficient to elicit apoptotic signaling [78], which includes immediate caspase-3 activation [79] leading to tau cleavage [80] and ultimately neuronal cell death. Significant increases in neurodegeneration have been reported following just a single day of ethanol exposure [23,32,35,63,69,81]. The cellular mechanisms of this pathway have recently been reviewed [22,78] and will not be discussed in further detail here. Gliosis and impairments of glial function are also consequences of ethanol exposure [82], and thus are additional channels through which anatomical effects potentially surface as maladaptive neurobehavior [83,84]. A very recently published study utilizing an adult-aged rat model of alcohol-induced brain damage reported positive gliosis marker detection as little as one day of ethanol exposure [85]. However, direct connections between gliosis and neurodegeneration has been shown to vary between cell type and brain region [86], and gliosis in the neonate exposure model apparently facilitates clearance of degenerating neurons [80]. Nevertheless, ethanol-induced gliosis may still ultimately contribute to the gross anatomical deformities and decreased mass that has been measured in a multitude of both human [87] and animal studies [78] of FASD. 

There are multiple implications for the long-term effects of anatomical changes of morphology and population numbers, because lasting deficits of neuronal circuit function and behavior in FASD models are likely due to early infrastructural changes in critically affected regions. It is important to consider how different brain regions and cell types are variably susceptible to ethanol-induced neurodegeneration when evaluating functional deficits characterized in FASD models [23,35,63,69]. Regions shown to be particularly susceptible to third-trimester equivalent binge-induced neurodegeneration include the cerebellum [88], hippocampus [35,69], olfactory bulb [89], occipital, cingulate and parietal cortices, caudate nucleus, nucleus accumbens, anterior thalamic nuclei and diagonal band of Broca [32,51]. Ethanol toxicity reaches beyond the immediate wave of neurodegeneration and dysmorphology, since secondary impacts on the migratory success and survivability of specific neuronal populations occur as well, and this can yield similar outcomes of long-term circuit dysfunction. Magnetic resonance imaging techniques performed in various animal models of FASD have illustrated gross anatomical size differences in many of the same regions listed above [90]. Gross anatomical differences in brain volume and shape may not be solely due to changes in cell population quantity, however. Altered neuronal process morphologies, spine density and soma size have also been observed as a result of binge exposure [76,91,92]. More recent methods in diffusion tensor imaging have enabled a more detailed live visualization of altered morphology of myelinated axons within white matter regions of human subjects diagnosed with FASD [93,94], thus allowing for even more anatomically focused approaches to developmentally relevant research and clinical attention.

Interneurons have been shown to decrease in numbers in the long term when exposed to ethanol in the third-trimester equivalent of early postnatal development [66,95,96]; however, see [97] for a study finding no differences in parvalbumin-positive neurons in other specific regions of the brain. As noted elsewhere in this review, there are physiologic ramifications [98] of a disproportional loss of inhibitory populations in other model systems of neurological disease [44,99,100,101] that have thematic relations to fetal alcohol neuropathology. Again, anatomical effects need not be restricted simply to a decrease in cell number, and likely include significant changes observed in dendritic morphology and cell-type organization within the physical locale of each affected circuit [95]. The significance of neuroanatomical changes observed in FASD models ultimately rely on correlative long-term functional changes. Neurobehavioral disease etiology as measured by functionality is incidentally dependent on the developmental timing of the binge exposure model used [102,103], and this variability is likely due to cell-specific courses of functional maturation within developing circuits. For instance, excitatory pyramidal cell activity arrives early in circuit development, where inhibitory synaptic function of GABAergic interneurons emerge relatively later in development (reviewed in [104]). A specified time of exposure means a signature profile of maturation and functionality of each cell type within a circuit. We attempt to address the physiological implications of the anatomical effects of early ethanol exposure below. 

### 4.2. Long-Lasting Physiological Effects of Acute Developmental Ethanol Exposure

Various assays of neuronal circuit function have been utilized to examine the long-term physiological impacts of early ethanol exposure, with the greatest emphasis on the hippocampus. Hippocampal structures are particularly sensitive to early ethanol binge-induced neurodegeneration and dysmorphology, and deficits in hippocampal-dependent memory are often a consequence. Appropriately, measurements of synaptic plasticity in the hippocampus have provided physiological confirmation of corresponding deficits in long-term potentiation [23,68,71,72,105]. These long-term outcomes were measured from rodents receiving binge-like exposure during early postnatal development, a human third trimester equivalent of brain development that experiences an explosion of synaptic refinement and fortification. The initial impact of ethanol toxicity exposure during this stage begins at least in part through NMDA receptor inhibition by ethanol. Excitatory neurons expressing NMDA receptors at their surface are particularly vulnerable to apoptotic neurodegeneration during early postnatal synaptogenesis [106]. A host of *in vitro* studies have demonstrated how ionotropic ligand-gated ion-channels are targets of ethanol toxicity [70,107]. This suggests that compromises of circuit development and long-lasting functionality are at least in part caused by ethanol interference with synaptic development as normally mediated through NMDA receptors [108,109]. 

Interestingly, acute low-dose ethanol treatment has been shown to actually protect cortical neurons in culture from excitotoxicity under low concentrations of exogenous NMDA [110,111]. Additionally, it has been hypothesized that inhibition of NMDA receptor activity by ethanol may explain observations in human ethanol-intoxicated head injury studies, where a limited range of exposure (low acute dose) decreased patient outcome severity [112]. In contrast, higher doses in the range of those produced in P7 rodent binge-like exposure studies do compound injury severity. Importantly, NMDA receptor antagonist-induced neurotoxicity in rats was found to be strictly age-dependent [113], with older adults unaffected at levels of exposure far exceeding those reported to be neurotoxic in embryonic and early-postnatal stages of development.

**Figure 1:**
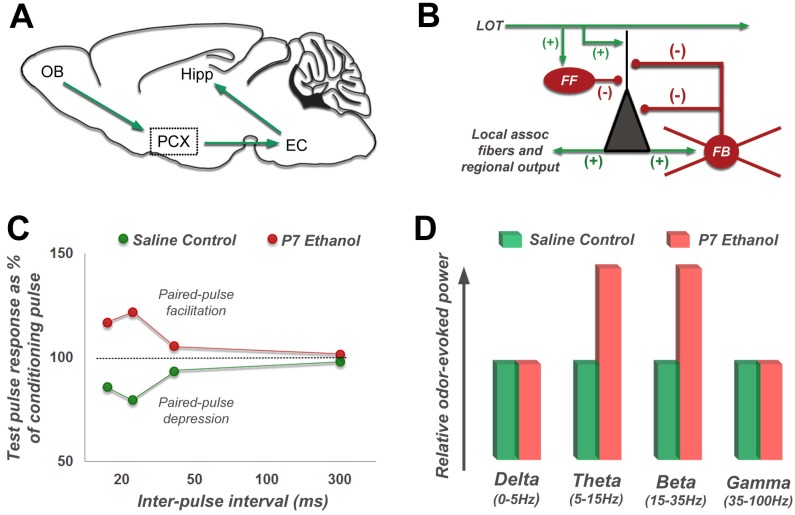
Physiological measurement of neural change in the olfacto-hippocampal pathway of adult mice treated with binge-like ethanol at postnatal day 7. (**A**) Diagram of primary information flow through the olfacto-hippocampal regional circuit. (**B**) Expansion of the piriform cortex (dotted box region in **A**) with basic local circuit feedback loop detail, including general modes of local connectivity existing between major cell types (green arrows = excitatory, red knobs = inhibitory). Stimulation from the olfactory bulb (OB) transmits to the piriform cortex (PCX) via the lateral olfactory tract (LOT). As pyramidal cells (grey triangle) are depolarized, associated feedback interneurons (FB) are activated to provide rapid inhibition of subsequent pyramidal cell activity, thus establishing temporally organized PCX processing and distribution of odorant information. (**C**) Idealized LOT-PCX paired-pulse analysis results (adapted from Sadrian *et al.* 2012 [23] and Wilson *et al.* 2011 [35]). Adults exposed to saline at P7 exhibit paired-pulse depression with reduced responses to the second test pulse at shorter inter-pulse intervals between the preceding condition pulse. In strong contrast, paired-pulse depression shifts to facilitation in adult mice exposed to ethanol at P7, suggesting dysfunctional local inhibition that is long-lasting. (**D**) Odor-evoked field potentials in the hippocampus were found to be enhanced in P7 ethanol-treated adult mice. Hyperexcitability or inhibitory deficits may also contribute to this type of interregional communication change along the olfacto-hippocampal pathway. Additional abbreviations: EC = entorhinal cortex, Hipp = Hippocampus, FF = feed-forward interneuron.

Two major foundations known to be impacted by ethanol: neuroanatomy and neurotransmission, are fatalistic determinants of long-term neuronal circuit function. This currently makes the precise etiology of ethanol-induced neuropathology a causality dilemma. As described above in Section-II, *in vivo* recordings of animal models with depth electrodes currently provide the most precise measurements of brain activity. We have recently reported significant long-lasting physiological effects *in vivo*, caused by binge-like ethanol delivered far earlier at postnatal day 7 [35]. Both local circuit function in the hippocampus and regional network activity in the olfacto-hippocampal circuit (olfactory bulb–piriform cortex–entorhinal cortex–hippocampus) were found to be hyperactive during both spontaneous resting states as well as during sensory-evoked responses (Figure 1). Cohort-treated mice showed a corresponding immediate wave of neurodegeneration in the hippocampus and other brain regions. In a follow-up study, we found the psychoactive drug lithium prevented both the initial wave of neurodegeneration and the corresponding neural circuit abnormalities otherwise lasting into adulthood [23]. Interestingly, in both these studies we found that long-term local circuit inhibition was dysfunctional, as assessed with paired-pulse analysis. P7 binge-exposed mice showed at adulthood a facilitated response to paired electrical stimulation of afferents to the piriform cortex rather than the standard depressed response seen in saline-exposed littermates. This shift from depression to facilitation may reflect loss of synaptic inhibition and/or changes in pre-synaptic transmitter release. Evidence for disinhibition was also present in inter-regional physiological measurements along the olfacto-hippocampal pathway. P7 ethanol-exposed mice recorded at three months old exhibited hyperexcitability in response to odor presentation in the piriform cortex and hippocampus [35]. All deficits described were corrected by co-treatment with lithium on the day of ethanol delivery [23]. Given that the psychoactive drug lithium prevents neurodegeneration, we hypothesize that this neuroprotective action spared inhibitory neurons and progenitor cells to result in a normally functional circuit. Lithium neuroprotection occurs at least in part through inhibition of caspase-3 mediated apoptotic signaling [24,25,32,79]. Equivalent findings in studies of peripheral nerve damage consequence in the spinal cord dorsal horn have demonstrated decreases in local inhibitory current following injury occur through a trans-synaptic apoptosis mechanism that is also caspase-dependent [114]. In this particular system the associated outcome of disinhibition is neuropathic pain, which serves as an analogous example of dysfunction resulting from unchecked sensory input. 

In development of many forebrain circuits, early postnatal stages experience high excitatory activity and low inhibitory activity, setting a stage for a sensitive period of plasticity. P7 binge exposure occurs during this developmental stage for areas like neocortex and the olfacto-hippocampal circuit, and illustrates the importance of excitatory/inhibitory balance and the timely vulnerability of inhibitory progenitors in this FASD model. During this period, synaptic connections between excitatory neurons become structurally and functionally refined with progressively adapted inhibitory cooperation of surrounding interneurons, resulting in optimized excitation/inhibition balance and circuit function. Therefore, early alcohol insult can hinder the regular establishment of a functional circuit during the sensitive period in a way that is developmentally unidirectional and permanent. It has been hypothesized that artificially returning a circuit to early developmental stages of excitatory/inhibitory dynamics through various interactive and pharmacological interventions could allow a return to the sensitive plastic period for reassignment of otherwise faulty connections toward a more properly adapted circuit [104]. 

Similar to our findings of long-term impairment in olfacto-hippocampal inhibitory activity following P7 ethanol [23], long-lasting changes in hippocampal excitability have been detected after adolescent ethanol exposure measured by sensory-evoked oscillations [36,115]. Therefore, this stage of development remains a window of plasticity during which structures remain vulnerable to cytotoxic insults that can destabilize subsequent circuit performance. Our observations in olfacto-hippocampal circuit dysfunction may be associated with the immediate wave of neurodegeneration observed throughout this circuit following acute ethanol exposure, as detected by caspase-3 and caspase-9a activation [116]. Just as with our studies, Criado and Ehlers [36] found that hippocampal event related oscillations in adult rats exposed to ethanol as adolescents were elevated at specific frequency ranges, again suggesting a possible shift in excitation/inhibition balance. The collective arguments from the studies described above and other related models described below help support the hypothesis that neurobehavioral dysfunction found in FASD is in large part that of faulty inhibition in local circuits and regional communication networks, which are impacted during early vulnerable stages from toxic insult to create lasting imbalance in synaptic excitation and inhibition.

Ethanol is a GABA receptor agonist and acute ethanol exposure directly enhances GABA release from interneurons in neonatal neocortex while the ethanol is present [117]. However, these immediate changes of inhibitory potential are not necessary to support a role for long-term excitation/inhibition imbalance in FASD etiology. Hypoactivation of NMDA receptors (as occurs under ethanol exposure) has been suggested to also contribute to inhibitory parvalbulmin-positive GABAergic interneuron dysfunction found in schizophrenia [118]. This population supports neural synchronization of oscillatory activity that is likely necessary for cognitive coherence [119]. Additionally, ethanol induced impairments of neurotransmission during critical periods of plasticity can perturb synaptic partner cell survival, by preventing normal activity-dependent synapse consolidation and/or pruning [120]. Importantly, the synaptic partners of these neurons can then die in turn as a result of input cessation. Although fewer in number, inhibitory interneurons make up a significant proportion of synaptic partnerships with pyramidal neurons in the CA1 region of the hippocampus [121]. Thus, through either direct [117] or indirect effects, ethanol exposure may ultimately impact interneuron survivability with functional consequences on inhibition. Additionally, latent neurogenesis and maturation of inhibitory interneurons persisting through the early postnatal stage means a greater number of inhibitory neurons that are affected in studies utilizing the early postnatal period of exposure [66], due to an unfulfilled proliferation potential of the progenitor population.

Prolonged developmental exposure to ethanol can impair odor discrimination [122]. In both studies we performed on olfacto-hippocampal physiology, gross discrimination between odors in ethanol-treated mice was found to be normal, despite the measured differences in local field potentials. The value of *in vivo* physiological measures becomes apparent here, because it may not always be possible to glean out behavioral changes within particular animal paradigms, yet there can be underlying disruptions in normal circuit function that are in fact significant. Such disruptions may be at the source of more complicated behavioral outputs currently carrying no observable animal model of behavior, but are particularly applicable to the nuanced neurobehavioral pathologies found in human FASD. In addition, pharmacological interventions guided toward FASD therapies can utilize *in vivo* physiological approaches to observe treatment effectiveness in real-time. With the advent of optogenetic methodology (reviewed in [123]), *in vivo* analysis and manipulation of circuits affected by early ethanol exposure quickly become boundless.

### 4.3. Long-Lasting Neurobehavioral Effects of Acute Developmental Ethanol Exposure

Each brain region, cell type and neuronal process impacted by ethanol exposure carries implications for subsequent measurable neurobehavioral deficits. The hippocampus is known to support spatial memory performance and experiences significant increases in neurodegeneration and decreased spine density after early ethanol exposure with a corresponding drop in spatial memory behavior task efficiency [23,35,69,73]. Unaided functional recovery of spatial memory impairment initially caused by binge ethanol has been reported [69]. This study measured no difference in compensatory neurogenesis after the initial wave of neurodegeneration was observed, thereby implying a mechanism of circuit reorganization (independent of replenishing cell numbers) that allowed behavioral recovery [106]. This would suggest that particular neurobehavioral pathologies are not irreconcilable to neuronal cell loss, or that the initial cell loss has no long-term impact on specific neurobehavioral integrity. However, we have found that if the initial wave of neurodegeneration was reduced or prevented by lithium co-treatment then ethanol-induced spatial memory impairment was prevented [23] implying the initial wave of ethanol-induced neurodegeneration may contribute in some manner to impairments in spatial memory performance initially observed in both studies. 

There have been many observations of long-lasting deficits in sensory system-driven behavior in both animals and humans as a result of early ethanol exposure [2,124,125]. At the same time, sensory processing disorders in humans have been postulated to impact the development of adaptive behavior [2,126], which can generally be described as successful daily survival skills, social interactability, and regular independence [127]. In relation to FASD neurobehavioral deficits, it is possible that disruption of normal neuronal circuit development carries consequences beyond the locale of physical toxic impact, and manifests as more than just a dysfunction in isolated behaviorally relevant circuits. As connections are established in broader networks of the brain during development, affected areas can impact those immediately unaffected via faulty adaptations in connective plasticity. In this light, all regions of the brain associated early on with sensory systems, known to be particularly affected in human FASD [128,129], become susceptible to perpetual dysfunction. We discuss below how other maladaptive behaviors are related to FASD, and provide possible insights for a mechanism of FASD as derived by common physiological measures of these related conditions.

## 5. Relationship of FASD to Other Disorders

There is an increasing awareness that an imbalance in synaptic excitation and inhibition are associated with a variety of psychopathologies across the lifespan [130,131,132], autism [3,133], schizophrenia [47,48,134], Alzheimer’s disease [44,135,136,137] and mood disorders [138]. One condition associated with excitatory/inhibitory imbalance of particular notoriety is epilepsy [139], because there is a high-comorbidity of epilepsy in human cases of FASD [5]. Despite the symptom diversity of these disorders, they are all associated with enhanced excitation/inhibition balance, most commonly mediated in part by a decrease in inhibitory synaptic function and/or loss of inhibitory GABAergic interneurons, although increases in membrane excitability can also occur [140,141]. Based on the data described above, the long-term consequences of developmental ethanol exposure now join this list of excitation/inhibition balance-related pathologies. Changes in excitation/inhibition balance are associated with disruption of synaptic homeostasis, synaptic plasticity and temporal synchrony of neural activity, all of which could impact perception, cognition, memory and general behavioral control.

Imbalances between excitation and inhibition may be particularly deleterious during early development. Changes in synaptic inhibition are associated with the opening and closing of developmental sensitive periods [131,142]. For example, newborn mammals, including humans, learn an attraction to the odor of their mother within hours after birth. In rodents, this early learning requires an association of olfactory stimulation with high levels of norepinephrine. The primary source of norepinephrine is the nucleus locus coeruleus (LC). During the perinatal period, inhibitory autoreceptors on LC noradrenergic neurons are reduced, while excitatory autoreceptors are enhanced. This results in a hyper-excitable LC precisely during the period that mother-infant attraction is most critical. As the infants mature and begin to explore beyond the nest, this balance of excitation and inhibition shifts towards greater inhibition (as generally described above), effectively ending this early sensitive period for odor learning [142]. In sensory cortical circuits such as the visual or auditory cortex, a similar inhibitory control over sensitive periods of plasticity occurs [131]. In this case, however, the control is mediated by GABAergic interneurons, especially those expressing the calcium binding protein parvalbumin. Enhanced activity in these neurons can help stop periods of elevated synaptic plasticity during early development [131]. Uncontrolled plasticity could result in aberrant circuit wiring, failure of normal dendritic and/or axonal pruning, and ultimate circuit dysfunction. Parvalbumin expressing GABAergic interneurons are generally fast spiking and form peri-somatic synapses onto principal neurons, making them very effective modulators of circuit excitability. Their activity may also be important for local circuit oscillations, especially in the gamma frequency range [98]. Loss or dysfunction of parvalbumin-expressing interneurons has been linked to schizophrenia [99], Alzheimer’s disease [44], epilepsy [100] and animal models of FASD [66,95,96,143] (though it should be noted that loss of parvalbumin expression does not necessarily signal cell loss [100]). 

Components of social behavior in autistic children have recently been compared to those in children with FASD [144,145]. Additionally, sensory processing deficits have been related to several autistic behaviors [126]. The behavioral deficits in autism are currently hypothesized to be at least in part the result of excitation/inhibition imbalance [45,46]. Studies in mice have recently shown how social behavior task performance can be influenced by either optogenetically [133] or pharmacologically [146] manipulating the existing excitation/inhibition balance (or imbalance) at specific brain regions. The commonalities of social behavior and sensory processing deficits found in autistic spectrum disorder and FASD, though arising from different origins, may point toward a general common mechanism that is the foundation for each neurobehavioral pathology.

## 6. Summary

Early developmental alcohol exposure has been shown to result in long-lasting neurobehavioral consequences. The precise etiology of reported deficits in animal models of FASD is by no means clear in any single paradigm. This is due to the multiple known modes of ethanol toxicity and the inherent complexity of brain development. Ultimate outcomes of early alcohol neuropathology, as measured by *in vivo* electrophysiology, reveal a disruption in local and regional activity in circuits that support behaviors coincidentally found to be impaired. Physiological alterations found in binge-exposure models of FASD involve decreases in inhibition at both local and regional circuit levels. As balance between excitation and inhibition is a critical foundation for normal neural circuit function, the disequilibrium observed in binge-exposure animal models of FASD illuminate a potential mode through which neurobehavioral deficits are generated. Imbalances (especially favoring reduced inhibition) have been associated with a variety of developmental disorders including Alzheimer’s disease and epilepsy. Direct and indirect sensitivity of parvalbumin expressing GABAergic interneurons to a variety of insults maybe a common factor among these diverse disorders, and an important direct or indirect target of early ethanol exposure.
